# Effect of Altered Retinal Cones/Opsins on Refractive Development under Monochromatic Lights in Guinea Pigs

**DOI:** 10.1155/2018/9197631

**Published:** 2018-02-20

**Authors:** Leilei Zou, Xiaoyu Zhu, Rui Liu, Fei Ma, Manrong Yu, Hong Liu, Jinhui Dai

**Affiliations:** ^1^Department of Ophthalmology, Eye and ENT Hospital, Fudan University, 83 Fenyang Road, Shanghai 200031, China; ^2^Key Laboratory of Myopia, Ministry of Health, 83 Fenyang Road, Shanghai 200031, China; ^3^Shanghai Key Laboratory of Visual Impairment and Restoration, Eye and ENT Hospital, Fudan University, 83 Fenyang Road, Shanghai 200031, China; ^4^Department of Ophthalmology, Jinling Hospital, School of Medicine, Nanjing University, 305 East Zhongshan Road, Nanjing 210018, China

## Abstract

**Purpose:**

To analyze the changes of refraction and metabolism of the retinal cones under monochromatic lights in guinea pigs.

**Methods:**

Sixty guinea pigs were randomly divided into a short-wavelength light (SL) group, a middle-wavelength light (ML) group, and a white light (WL) group. Refraction and axial length were measured before and after 10-week illumination. The densities of S-cones and M-cones were determined by retinal cone immunocytochemistry, and the expressions of S-opsins and M-opsins were determined by real-time PCR and Western blot.

**Results:**

After 10-week illumination, the guinea pigs developed relative hyperopia in the SL group and relative myopia in the ML group. Compared with the WL group, the density of S-cones and S-opsins increased while M-cones and M-opsins decreased in the SL group (all, *p* < 0.05); conversely, the density of S-cones and S-opsins decreased while M-cones and M-opsins increased in the ML group (all, *p* < 0.05). Increased S-cones/opsins and decreased M-cones/opsins were induced by short-wavelength lights. Decreased S-cones/opsins and increased M-cones/opsins were induced by middle-wavelength lights.

**Conclusions:**

Altered retinal cones/opsins induced by monochromatic lights might be involved in the refractive development in guinea pigs.

## 1. Introduction

Myopia is the most common ocular disorder that causes visual dysfunction such as premature cataracts, glaucoma, retinal detachment, and macular degeneration. The prevalence of myopia is increasing, while there has not been a breakthrough in the prevention and treatment of myopia because the underlying mechanisms have not fully been understood.

Previous studies have suggested that emmetropization depends on visual information [1, 2]. When form deprivation is employed to reduced contrast and spatial frequency content of animals, myopia is induced [3–5]. Negative lens wearing which affects the light sense also results in myopia [6–8]. Therefore, color sense, as another important characteristic of vision in mammals, may also be involved in emmetropization. A survey of epidemiology showed that the prevalence of myopia is significantly lower in the students with color vision deficiencies than in those with normal color vision [9]. The authors speculated that this phenomenon might be linked to reduced functionality of the L/M chromatic mechanism. Kröger and Wagner [10] found that nasotemporal diameters of fish, the blue acara, were enlarged when they were raised in lights of longer wavelength. When chickens were raised in blue or red light for two days, the chickens in red light became 1.69 D more myopic during the subsequent rearing period compared with chickens raised in blue light [11]. All these studies suggest that emmetropization can be affected when the chromatic cues are modified, though the mechanism is still unclear.

There are two kinds of photoreceptors in the retina: rods and cones. The latter mediate color vision [12]. Furthermore, in foveate animals, high spatial resolution is mediated by cone vision. Mice had grating acuity when lacking only functional rods but had no detectable grating acuity when lacking both rods and cones [13]. Two or more types of cones with spectrally different visual pigments (opsin binds to 11-cis retinal) are required to generate color discrimination. In the outer segment (OS), visual pigments absorb different wavelengths of light. In retina of primates, there are three types of functional cones, which contain long-wavelength-, medium-wavelength-, or short-wavelength-specific opsin in OS [14, 15]. However, other mammals such as guinea pigs have only two types of cones that respond maximally to two different wavelengths: S-cones respond to 430 nm short-wavelength light while M-cones respond to 530 nm middle-wavelength light [16]. In these mammals, cones have a characteristic distribution in retina. It has been established that in guinea pigs, the ventral retina expresses mostly M-cones while the dorsal retina expresses mostly S-cones.

The expression of opsin in cones in retina is not constant. Long-term changes in the spectral composition of light, as typical for the transition from dusk to dawn, or changes in the spectral transmittance of water at different depths, can alter the expression of levels of opsins and cones [17]. Previous studies showed that the expression of M-cones and opsins in guinea pigs increased in 530 nm wavelength light [18]. This observation shows that color processing in the retina displays developmental plasticity. It is also expected that the density of cones changes with axial length and refractive state [19]. In guinea pigs with form deprivation or negative lens-induced myopia, the expressions of S-opsin mRNA increased. Retinal cones serve as detector for form deprivation and defocus [20]. Therefore, changes in the expression of the cone opsins may play a role in the development of experimental myopia.

The study done by Hu et al. [18, 20] indicated that the number and distribution of M-cones changes with monochromatic light. In our research, we further investigated the relationship between changes of cone densities and refractive development in monochromatic light. The aim of the present study is to determine how long-term monochromatic environments affect the expression of cones and opsins and whether the changes of refraction are related to the metabolism of the cones in retina under these environments in guinea pigs. Our research may enhance the understanding of mechanism between color sense and myopia and may bring forward a novel way to prevent myopia.

## 2. Materials and Methods

### 2.1. Animal Model

All research procedures were approved by the Institutional Animal Care and Ethics Committee at the Eye and ENT Hospital of Fudan University and were in compliance with the ARVO Statement for the Use of Animals in Ophthalmic and Vision Research.

Sixty pigmented guinea pigs (2 weeks old) from the Animal Experiments Laboratory (Fudan University, Shanghai, China) were randomly assigned to three groups. In the SL group (*n* = 20), the guinea pigs were raised under 430 nm short-wavelength lights for 10 weeks. In the ML group (*n* = 20), the guinea pigs were raised under 530 nm middle-wavelength lights. In the WL group (*n* = 20), the guinea pigs were reared in normal lights without any intervention. Three types of LED light tubes were used: short-wavelength light (blue light, peak value of 430 nm, half-bandwidth of 10 nm), middle-wavelength light (green light, peak value of 530 nm, half-bandwidth of 10 nm), and normal light (white light, broadband, color temperature of 5000 K). The photon flux density of each treatment was selected to produce equal quantal numbers for each group and was set at 3 × 10^−4^ *μ*mol·cm^−2^·s^−1^ (about 1770 mW·m^−2^ for blue light, 700 mW·m^−2^ for green light, and 740 mW·m^−2^ for white light). Specially designed rearing cages of the three groups were mutually independent, and different wavelength lights did not interfere with each other. Details on the cage and light settings were described previously in Materials and Methods [21]. All the animals were raised under a 12/12 h light/dark cycle.

### 2.2. Refraction Assessment

Refraction, corneal curvature (CC), anterior chamber depth (ACD), lens thickness (LT), and axial length (AL) were measured at the onset of the experiment and 10 weeks later in the guinea pigs. Data of both eyes were enrolled. Refraction was measured with retinoscopy in a dark room. One hour before which, a drop of 1% cyclopentolate hydrochloride (Alcon, Belgium) was topically administered to achieve cycloplegia. Measures were performed by an experienced investigator who was blinded to the group assignment. The results of refractive states were recorded as the mean refractions in the horizontal and vertical meridians. The corneal radius of curvature was measured by keratometry (Topcon OM-4, Japan). The animals were anesthetized with a topical application of 4% oxybuprocaine hydrochloride, and then the ACD, LT, and AL were measured by A-scan ultrasonography (11 MHz; Optikon HiScan A/B). More details for the specific instruments and methods for these biometric measurements were described in our other published article [22].

### 2.3. Immunohistofluorescence

After 10-week raising, the guinea pigs were executed by cervical vertebra dislocation at 8 o'clock. Ten animals were randomly selected from each group. The retinas that marked at the 12 o'clock position by a notch were dissected in ice-cold PBS and immersed in 4% paraformaldehyde for 20 min. The left and right retinas were rinsed in PBS for three times and were prepared for immunocytochemistry of S-cones and M-cones, respectively.

The left retinas were then exposed to polyclonal antibodies specific to the S-opsin (rabbit anti-S-opsin; Chemicon, USA) at a 1 : 200 dilution. The retinas were then incubated with the primary antibodies overnight, rinsed for 5 times, and then incubated for 1 h in a dark chamber at 37°C with secondary antibodies (goat anti-rabbit IgG conjugated FITC; Molecular Probes, USA) at a 1 : 100 dilution, rinsed for 5 times. The right retinas were exposed to primary polyclonal antibodies specific to the M-opsin (rabbit anti-M-opsin; Chemicon, USA) at a 1 : 200 dilution and a secondary antibody (goat anti-rabbit IgG conjugated rhodamine; Molecular Probes, USA) at a 1 : 100 dilution at the same procedure.

A scanning laser confocal microscope was used to photograph S-cones (green florescence) in the left eyes and M-cones (red florescence) in the right eyes. The dorsal retina measured 4 × 2 mm. It was measured from 2 mm vertically above the optic disc, and laterally, it was measured 2 mm away from the optic disc on both sides. The ventral retina was similarly measured, but this was done on the opposite side (vertically below the optic disc). To examine the topographic distribution of the fluorescent cones, the numbers of fluorescent cones were counted in contiguous sampling windows (240 × 132 *μ*m window) from one side to the other side. The cone density data were analyzed with Image-Pro Plus v5.1 software.

### 2.4. Real-Time PCR

Total RNA was isolated from the left eye of ten animals using the phenol-chloroform extraction method of Chomczynski and Sacchi [23]. The following primers obtained from Hu et al. [18] were used: *β*-actin, forward 5′-GACGAAGCCCAGAGCAAA-3′, reverse 5′-CCAGAGGCATACAGGGACAG-3′; S-cone, forward 5′-GAGTATTTCGCCTGGTTCCTT-3′, reverse 5′-CCTTCTGGGTTGTAGCTGATT-3′; M-cone, forward 5′-TCATCGCATCCATCTTTACCA-3′, reverse 5′-AGCACGAAGTAGCCGTAGACC-3′. PCR conditions were performed as follows: 3 min preincubation at 95°C followed by 40 cycles of 30 s at 95°C, annealing at 58°C for 30 s, and extension at 72°C for 30 s. PCR products were verified by melting curve analysis, agarose gel electrophoresis, and DNA sequencing. All experiments were performed at least three times. Expression of the target mRNAs was normalized to *β*-actin levels, and the 2^−ΔΔCT^ (cycle threshold) method was used to calculate relative expression levels. The results of real-time PCR were reported as the fold changes in gene expression levels and checked by analyzing melting curves.

### 2.5. Western Blot

Total protein was extracted from frozen retinas of the right eye of ten animals with ice-cold extraction buffer as well as protease inhibitors. After calculating the protein concentrations, samples (30 *μ*g) were electrophoresed and subjected to Western blotting using S-cone and M-cone antibodies. The membranes were incubated overnight with primary antibodies at a 1 : 100 dilution (rabbit anti-guinea pig S-cone; Abcam, Cambridge, UK) and a 1 : 500 dilution (chicken anti-guinea pig M-cone; Millipore, Massachusetts, USA) at 4°C in blocking solution. The membranes were then washed three times with TBST and incubated with secondary antibodies for another 1 h at room temperature. Goat anti-rabbit IgG-HRP was for S-cone at a 1 : 3000 dilution (Abmart, Shanghai, China), and rabbit anti-chicken IgY (IgG) (H + L) was for M-cone at a 1 : 1000 dilution (Jackson ImmunoResearch, Pennsylvania, USA). After washing, the membranes were stained with an ECL kit (Thermo Fisher Scientific, USA). Images were captured with a Fujifilm LA-S3000 imaging system and analyzed with MultiGauge software (Fujifilm, Japan). The band of each protein was normalized by *β*-tubulin (Kang Chen, China) as an internal control.

### 2.6. Statistical Analysis

Statistical analysis was performed using SPSS 15.0 statistical software (IBM, Chicago, IL). All values are shown as the mean ± standard deviation (SD). A one-way ANOVA was used for comparisons of the three groups. All pairwise comparisons were adjusted for multiple comparisons using the Bonferroni approach. A difference at *p* < 0.05 was considered statistically significant.

## 3. Results

### 3.1. Biometric Changes in the Guinea Pig Eyes Induced by Monochromatic Lights

**Figure 1:**
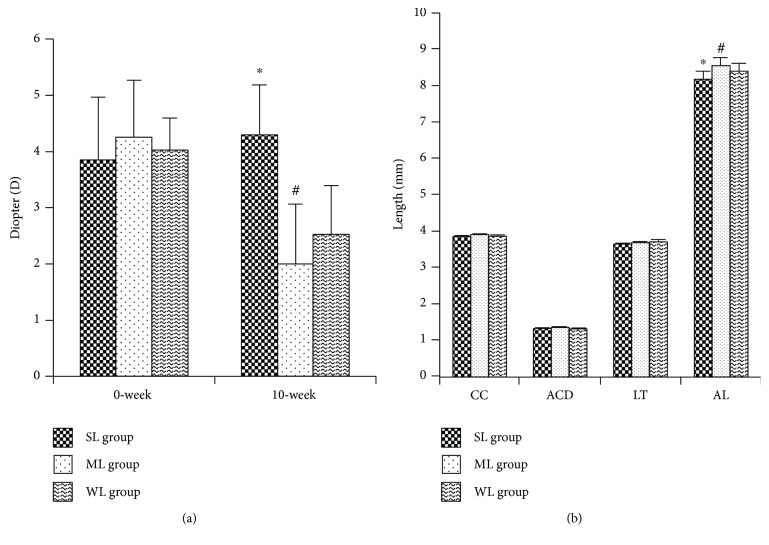
Biometric changes before and after intervention. Mean biometric results from guinea pigs reared under the three different lighting conditions (SL: short-wavelength light; ML: middle-wavelength light; WL: white light). (a) Diopters and (b) biometric results in 0-week and 10-week in the three groups. AL: axial length. ∗ means significant differences between the SL and WL groups. # means significant differences between the ML and WL groups.

There were no significant differences in refraction, CC, ACD, LT, or AL among the three groups of guinea pigs at the beginning of the experiments. After ten weeks, the mean sphere refraction in the SL group was significantly more hyperopic than that in the WL group (1.95 D; +4.30 ± 0.88 D versus +2.53 ± 0.86 D; *n* = 20, *p* < 0.01), while refraction in the ML group was more myopic than that in the WL group eyes (0.75 D; +2.00 ± 1.06 D versus +2.53 ± 0.86 D; *n* = 20, *p* < 0.01). The AL of the SL group was shorter relative to that in the WL group by 0.26 mm (8.17 ± 0.11 mm versus 8.40 ± 0.20 mm; *n* = 20, *p* < 0.01), while ML eyes were longer relative to the WL eyes by 0.12 mm (8.56 ± 0.20 mm versus 8.40 ± 0.20 mm; *n* = 20, *p* < 0.01). Other parameters, such as CC, AC, and LT, showed no significant differences among the three groups (Figure 1).

### 3.2. Densities of Retinal Cones Were Changed in Monochromatic Lights

**Figure 2:**
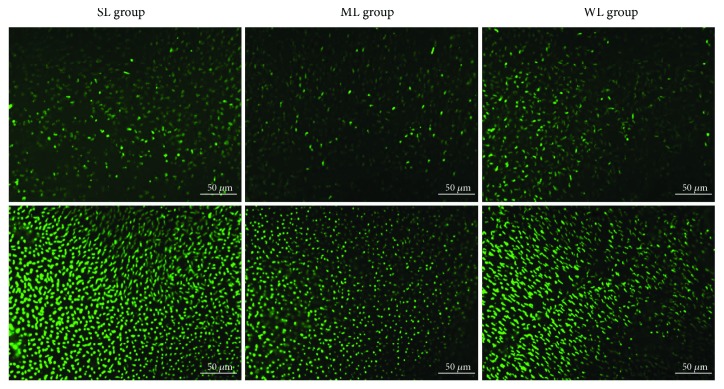
S-cones changed in different areas in the three groups. Top: dorsal retina; bottom: ventral retina. SL: short-wavelength light; ML: middle-wavelength light; WL: white light. Scale bar = 50 *μ*m.

**Figure 3:**
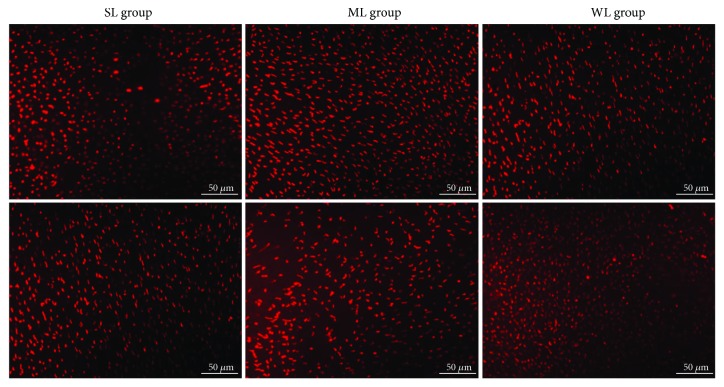
M-cones changed in different areas in the three groups. Top: dorsal retina; bottom: ventral retina. SL: short-wavelength light; ML: middle-wavelength light; WL: white light. Scale bar = 50 *μ*m.

Because the dorsal retina was reported to be dominated by M-cones, whereas the ventral retina was dominated by S-cones, we studied the abundancy and opsin expression of the two types of cones in the dorsal and ventral retinas. After 10 weeks, retinal cone immunocytochemistry indicated that, compared with the WL group, the S-cone density increased and the M-cone density decreased in the dorsal and ventral retinas of the SL group (all, *p* < 0.05) and the density of S-cone decreased and the density of M-cone increased in the dorsal and ventral retinas of the ML group (all, *p* < 0.05) (Figures 2 and 3 and Table 1).

### 3.3. S- and M-Opsins Were Changed at mRNA and Protein Expression Levels (Fold Changes)

**Figure 4:**
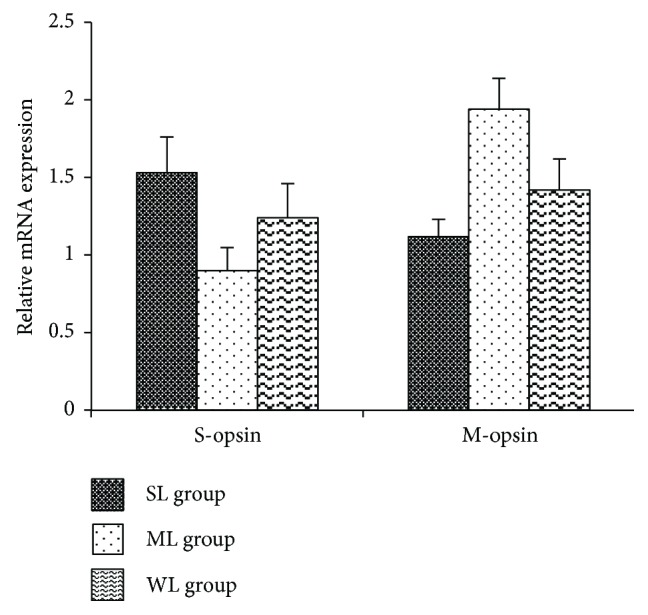
S- and M-opsin mRNA expressions in the retinas of guinea pigs irradiated by short-wavelength light, middle-wavelength light, or white light (represented as a bar graph). Expressions of S-opsins in the retinas of guinea pigs in the SL group were significantly higher than those in the ML group (*p* < 0.05) while expressions of M-opsins in the retinas of guinea pigs in the SL group were significantly lower than those in the ML group (*p* < 0.05). SL: short-wavelength light; ML: middle-wavelength light; WL: white light. ∗ means significant differences between the SL and WL groups. # means significant differences between the ML and WL groups.

Real-time PCR showed that, after 10 weeks, relative S-opsin mRNA levels in the retinas of the SL, ML, and WL groups were 1.53 ± 0.23, 0.90 ± 0.15, and 1.24 ± 0.22, respectively; relative M-opsin mRNA levels in the retinas of the SL, ML, and WL groups were 1.12 ± 0.11, 1.94 ± 0.2, and 1.42 ± 0.2, respectively. Compared with the WL group, the expression of S-opsin was increased (*p* < 0.05), while M-opsin expression was decreased (*p* < 0.05) in the SL group, and the expression of S-opsin was decreased (*p* < 0.05), while M-opsin expression was increased (*p* < 0.05) in the ML group (Figure 4).

**Figure 5:**
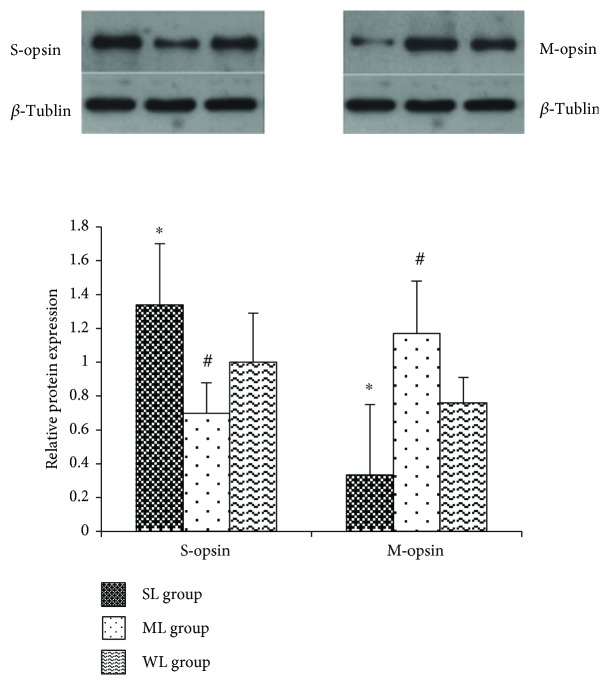
S- and M-opsin protein expressions in the retinas of guinea pigs illuminated by short-wavelength light, middle-wavelength light, or white light (represented as a bar graph). Expressions of S-opsins in the retinas of guinea pigs in the SL group were significantly higher than those in the ML group (*p* < 0.05) while expressions of M-opsins in the retinas of guinea pigs in the SL group were significantly lower than those in the ML group (*p* < 0.05). SL: short-wavelength light; ML: middle-wavelength light; WL: white light. ∗ means significant differences between the SL and WL groups. # means significant differences between the ML and WL groups.

Western blot analysis also showed that changes in the levels of S-opsins and M-opsins in the retina differed according to illumination at different wavelengths of light. After 10 weeks, relative S-opsin protein levels in the retinas of the SL, ML, and WL groups were 1.34 ± 0.36, 0.70 ± 0.18, and 1.00 ± 0.29, respectively; relative M-opsin protein levels in the retinas of the SL, ML, and WL groups were 0.34 ± 0.41, 1.17 ± 0.31, and 0.76 ± 0.15, respectively. Compared with the WL group, the expression of S-opsin was increased (*p* < 0.05), while M-opsin expression was decreased (*p* < 0.05) in the SL group, and the expression of S-opsin was decreased (*p* < 0.05), while M-opsin expression was increased (*p* < 0.05) in the ML group (Figure 5).

## 4. Discussion

Chromatic cues may normally contribute to the regulation of refractive development [24–26]. Our previous study also indicated that ocular refraction and axial length of the eyes of animals adjusted refractive states according to the wavelength of light. Compared to animals exposed to white lights, animals in mid-wavelength light become more myopic and animals in short-wavelength light become more hyperopic [21, 22, 27]. In this study, we selected guinea pigs aged 2 weeks, raised them under 430 nm or 530 nm monochromatic lights for 10 weeks, and examined the longitudinal changes in refraction and eye growth at the end the 10th week. The eyes from the WL group displayed a decrease in hyperopic error from approximately 4.03 D to 2.53 D, accompanied by an elongation of the axial length from 7.57 mm to 8.40 mm. Compared to the WL group, the eyes in the SL group demonstrated more hyperopia by 1.95 D and a shorter axial length by 0.26 mm. Meanwhile, the eyes in the ML group showed a further reduction of hyperopic refractive error (0.75 D) and greater axial length (0.12 mm). For all these three groups, corneal curvature showed a similar trend of change during the experiment, regardless of wavelength of lights they were exposed to. The same were true of anterior chamber and lens thickness. This result is consistent with the results in fish [28] and chicks [11].

Studies of refractive development under monochromatic lights vary in different animals such as monkeys [29], tree shrews [30], chicks [11], and guinea pigs [31]. Recent investigation in tree shrews reported that chronic exposure to long-wavelength lights (628 ± 10 nm) produced hyperopic shifts, but in guinea pigs in our study (530 ± 10 nm), the refractions changed in the opposite direction. Even for the same animal exposed in long-wavelength light, experimental results of refractive state can be surprisingly opposite in different researches. The results of Smith et al. [32] also differ significantly from the findings of Liu et al. [29] in rhesus monkeys under long-wavelength lights. Why do the patterns of results obtained in these studies differ? There are substantial methodological differences between the studies, such as age of the subjects, sources of lights (LED versus long-wavelength pass filter), and luminance levels. These differing results demonstrate clearly that we do not yet understand all relevant parameters that determine the effects of light of different spectral composition on refractive development.

The mechanism for how monochromatic light controls eye growth is not well understood. Previous studies found that refractive development depends on the wavelength of the illuminating lights. Natural light, as a mixture of different monochromatic lights with different wavelengths, may contribute to a backward displacement of the retina toward the eye's image plane, causing longitudinal chromatic aberration (LCA) [33]. LCA is a wavelength-dependent refractive error that influences the emmetropization of the eyes. For an emmetropic human eye looking at a distant object, the focal image for each wavelength is usually formed at different locations, with short wavelengths focused in front of the retina, long wavelengths behind the retina, and middle wavelengths at the retina [9]. Therefore, LCA was initially considered as the reason for abnormal refractive development under monochromatic environment.

In our previous research [22], the difference of the refraction between the animals raised under 530 nm wavelength lights for 12 weeks and counterparts under 430 nm wavelength lights was 4.5 D, while LCA between these two lights was only 1.5 D. In our study, after 10 weeks under monochromatic lights, refractive changes were also larger than those required to compensate for LCA. The magnitudes of refractive changes did not agree well with prediction by the LCA in the guinea pigs' eyes. Therefore, factors other than chromatic defocus must be involved to explain the overcompensated refraction induced by monochromatic lights.

As photoreceptor cells for bright light and color vision, retinal cones may be related to ocular growth [15, 34]. Short-wavelength sensitive (S) cones (maximum absorbance, 430 nm) dominate in the ventral parts of the retina in guinea pigs while middle-wavelength sensitive (M) cones (maximum absorbance, 530 nm) in the dorsal regions [35, 36]. The transitional zone between these two retinal areas is populated by coexpressing cones that express both S-cone and M-cone photopigments [35]. In our study, we used blue lights and green lights with peak sensitivities at 430 nm and 530 nm, respectively [36]. Lights at short wavelength and middle wavelength will mainly be absorbed by the S-cones in the ventral part and M-cones in the dorsal part of the retina, respectively. Thus, it was convenient to compare the changes of cones and opsins in the ventral and dorsal parts under different lighting conditions in this study and further investigate the relationships of cones and refractions in the eyes of guinea pig.

The results of this study and the previous studies indicate that refractive error changes are larger than those required to compensate for LCA. We speculate that this may be related to the changes in retinal cones and opsins. Lighting environments have influence on number and distribution of cones, and such influence is associated with changes in expression of opsins [18]. For example, winter flounders have only one cone pigment, but during metamorphosis to benthic, they express three other cones [37]. Mantis shrimps are sensitive to the wavelengths of lights in the environment because these shrimps adjust the properties of their cone filters [38]. Our study also indicated that, compared with the WL group, the S-cone density increased and the M-cone density decreased in the dorsal and ventral retinas of the SL group, while the reverse was true of the ML group. Results of the study done by Hu et al. indicated that coexpressing cones (cones that express both S-cone and M-cone photopigments) in the transitional zone of the guinea pig retina could regulate the number of S-cones and M-cones in the retina [18]. Thus, cones expressed in the transitional zone are probably related to or identical in origin with S-cones and M-cones and can lead to plasticity in different monochromatic environments.

Although in both our study and the research done by Hu et al., M-cones displayed changes with monochromatic lights, S-cones showed no change in number and distribution. In our study, the density of S-cones changed under different monochromatic lights, whereas in the study by Hu et al., S-cones were not affected in monochromatic lights [18]. The different response of S-cones may be explained by different short-wavelength lights used and duration of lighting in these two studies. Hu et al. [18, 20] used violet light whose peak value was at 400 nm, whereas blue light at 430 nm was chosen in our study. Besides, the modeling time in this research was 2 weeks longer than Hu's research, which may also cause the different results.

As the key components of cones, opsins were studied as well to see if there were any changes in expression with spectral environment. We compared expressions of S-opsins and M-opsins on the levels of mRNA and protein and found the changes in cones match those in opsins. The increase in S-cone and M-cone densities led to an increase in S-opsin and M-opsin expressions and vice versa.

In our study, opsin expression went up when the preferred wavelength was predominant but the biological sense is still unknown. We hypothesize that change of visual by monochromatic light is to provide cues for eye growth. The visual may trigger the changes in cone density and opsin expression that finally affect eye growth. For one thing, retinal cones may affect ocular growth of guinea pigs by responding to different defocusing signals under monochromatic light. Former studies have hinted that the short-wavelength sensitive cones respond to the myopic defocus and the longer-wavelength sensitive cones respond to the hyperopic defocus [33]; for another, the changes in cones and opsins under monochromatic lights may activate relative signaling pathways by local regulation. Under monochromatic lights, the metabolites of opsins, such as retinoic acid and other factors in the retinoic acid cycle, may induce the retina to secrete a signal that modulates the related factors. These factors in turn modulate eye growth, thereby overcompensating for defocusing of the LCA.

The limitation of this paper is that the causal relationship between the change of cone expression and refractive development remains unclear. The alteration in cone opsin expression might be associated with the circumstantial change of the monochromatic lights. Hopefully, future progress could be made by pharmacologically changing S and M cone opsin expressions in eyes of guinea pigs. In such an interference model under monochromatic lights, refractive development can be further investigated.

In summary, retinal cones and opsins changed under monochromatic lights and might play a very important role in ocular growth. Further research is needed to study the function of metabolic products of retinal cones and investigate the roles of cones and opsins in eye growth.

## Figures and Tables

**Table 1:** The densities of S-cones and M-cones in the different groups (mean ± SD, mm^2^).

Cell	Area	SL group		ML group		WL group
		Mean ± SD	*p*	Mean ± SD	*p*	Mean ± SD
S-cone	Dorsal	1476 ± 317.5	0.014	592 ± 105.8	0.018	887.7 ± 90.2
	Ventral	17844 ± 518.7	0.026	11766 ± 108.3	0.029	15621 ± 185.6
M-cone	Dorsal	12646 ± 554.7	0.041	18115 ± 761.4	0.020	16492 ± 835.3
	Ventral	4646 ± 737.2	0.022	8494 ± 817.6	0.011	5992 ± 635.9

“*p*” represents the significance of the differences between the eyes from the SL and WL groups or the ML and WL groups. SL: short-wavelength light; ML: middle-wavelength light; WL: white light.
